# An Analytic Model of Tissue Self-Healing and Its Network Implementation: Application to Fibrosis and Aging

**DOI:** 10.3389/fphys.2020.583024

**Published:** 2020-10-29

**Authors:** Béla Suki, Jacob Herrmann, Jason H. T. Bates

**Affiliations:** ^1^Department of Biomedical Engineering, Boston University, Boston, MA, United States; ^2^Department of Medicine, The University of Vermont, Burlington, VT, United States

**Keywords:** repair, agents, network, fixed point, random walk

## Abstract

Here we present a model capable of self-healing and explore its ability to resolve pathological alterations in biological tissue. We derive a simple analytic model consisting of an agent representing a cell that exhibits anabolic or catabolic activity, and which interacts with its tissue substrate according to tissue stiffness. When perturbed, this system returns toward a stable fixed point, a process corresponding to self-healing. We implemented this agent-substrate mechanism numerically on a hexagonal elastic network representing biological tissue. Agents, representing fibroblasts, were placed on the network and allowed to migrate around while they remodeled the network elements according to their activity which was determined by the stiffnesses of network elements that each agent encountered during its random walk. Initial injury to the network was simulated by increasing the stiffness of a single central network element above baseline. This system also exhibits a fixed point represented by the uniform baseline state. During the approach to the fixed point, interactions between the agents and the network create a transient spatially extended halo of stiffer network elements around the site of initial injury, which aids in overall injury repair. Non-equilibrium constraints generated by persistent injury prohibit the network to return to baseline and results in progressive stiffening, mimicking the development of fibrosis. Additionally, reducing anabolic or catabolic rates delay self-healing, reminiscent of aging. Our model thus embodies what may be the simplest set of attributes required of a spatiotemporal self-healing system, and so may help understand altered self-healing in chronic fibrotic diseases and aging.

## Introduction

Self-healing is the ability for spontaneous repair following injury and is a critical homeostatic feature of biological systems that allows them to survive the rigors of life’s experiences for extended periods. Biological self-healing involves a complex interplay between numerous cell types in the body (Carlson and Longaker, 2004; Vidmar et al., 2017), leading inexorably to complete resolution of whatever damage the injury caused (Mutsaers et al., 1997). Being able to harness the power of self-healing is thus of great importance for medicine, and it has even recently led to the bio-inspired development of artificial self-healing systems (Mutsaers et al., 1997; Hong et al., 2019). On the other hand, aberrant self-healing is also responsible for many chronic pathologies that at best leave behind an inconveniently remodeled tissue, and at worst can be progressive and eventually fatal. An example is idiopathic pulmonary fibrosis (IPF), an insidious disease typically associated with aging that has a median survival of 3.8 years from diagnosis (Raghu et al., 2014). Nevertheless, we still have a poor understanding of the essential components required of any system in order for it to exhibit the property of self-repair; therefore, how repair becomes dysregulated in a disease such as IPF remains a mystery.

What are the general features of self-healing systems necessary for successful repair? We propose that there are three: (1) the ability to detect injury and initiate local reparative processes in response, (2) the availability of sufficient material and energy reserves to complete the repair, and (3) the ability to cease reparative processes when injury is no longer present. In biological systems, all of these features are associated with various cell types together with certain properties of the extracellular matrix (ECM). All are necessary for self-repair to proceed efficiently. In the absence of damage detection, for example, reparative processes would have to be initiated at random locations throughout a tissue. This is much less efficient than targeted self-healing, as has been demonstrated, for example, with respect to the elimination of fibrotic lesions from the lung parenchyma (Suki et al., 2007). Likewise, the availability of resources is obviously critical if damage is to be repaired at all, and the ability to turn off the anabolic processes involved in repair when they are no longer needed is essential if chronic remodeling and inflammatory pathologies are to be avoided. Evolution tends to favor efficiency, so it is not surprising that the above three features are exhibited ubiquitously throughout the animal kingdom.

Here we focus on the repair of damaged ECM, and how it might become deranged in chronic fibrotic disease and aging. The autonomous self-healing of damaged lung parenchyma requires that the intactness of the ECM be constantly assessed by migrating cells. When such cells detect ECM fragments they can either initiate wound healing directly (Tolg et al., 2014) or release chemotactic factors to attract inflammatory cells that orchestrate the digestion and removal of damaged tissue (Nakagawa et al., 1999). Fibroblasts must then be recruited and activated to lay down new ECM in order to replace the tissue that has been lost (Hartupee and Mann, 2016). Subsequent changes in cross-linking between protein fibers then serve to stabilize the mechanical structure of the new tissue (Rief et al., 1997; Thompson et al., 2001). The final step, recognizing when repair is complete, is the least well understood, but likely involves a highly elaborate cell-cell signaling scheme that brings the inflammatory cascade to an end (Nathan, 2002). These are all functions that require a large number of cells and their products to function as a cohesive system within which self-healing arises as an emergent property.

The goal of this study is to develop a mathematical model of homeostatic ECM maintenance that is minimal in the sense of representing the processes outlined above in a general sense without undue regard for their precise details. Our premise is that this will abstract the general dynamic processes involved in self-repair of tissue and then provide a platform for investigating its breakdown in aging and the pathogenesis of chronic fibrotic disease.

## Model Description

We first develop a simple 2-dimensional non-linear representation of biological soft tissue characterized by a local state variable, tissue stiffness of the ECM, and an activity variable representing a single cell interacting with the ECM. We find the fixed point of this system and analyze its stability. We then implement the equations of this model in an elastic network mimicking the mechanical properties of a macroscopic section of tissue and populate the network with discrete agents representing the cells that take part in tissue repair. These agents carry out the self-healing maintenance program as they migrate over the network by modulating the mechanical properties of the network members. By maintaining the system under non-equilibrium conditions to represent a persistent insult to the tissue, and altering model parameter values from baseline to represent pathological processes, we demonstrate both how fibrosis can develop and how self-healing can degrade with aging.

### A Non-linear Dynamic Model of Self-Healing

Consider a single agent at a fixed location on a sheet of tissue. The agent is characterized by a single state variable, *a*′, representing its activity level, while the patch of tissue upon which it sits is characterized by another state variable, *k*′, representing the local stiffness of the tissue. Both *a*′ and *k*′ are functions of time, *t*′.

We assume that cellular activity modulates tissue stiffness, which in turn regulates cellular activity, so

(1) $$\frac{d\,a^{\prime }}{d\,t^{\prime }}=f\,\left(a^{\prime },k^{\prime }\right)$$

(2) $$\frac{d\,k^{\prime }}{d\,t^{\prime }}=g\,\left(a^{\prime },k^{\prime }\right)$$

where *f* and *g* are continuously differentiable non-linear functions. In the special case that the agent does not significantly affect tissue properties, the stiffness will remain essentially constant, and we let the activity of an activated cell decay exponentially toward a steady-state baseline level of β with rate-constant α. That is,

(3) $$\frac{d\,a^{\prime }}{d\,t^{\prime }}=\alpha \,\beta -\alpha \,a^{\prime }$$

The solution to Eq. 3 is

$$a^{\prime }\,\left(t^{\prime }\right)=\beta +W\,e^{-\alpha \,t^{\prime }}$$

where *W* is a constant determined by the initial conditions. Note that as *t*′→∞, the solution approaches β at an exponential rate α, which is independent of *W*.

In the more general case, where the activation state of the agent does significantly affect tissue properties, we assume that the steady-state activation level, β, increases with the stiffness, *k*′. However, activation cannot increase indefinitely because it requires energy, whereas tissue stiffness is a passive property that is less constrained in this regard. We therefore let the dependence of activation on stiffness exhibit first-order saturation behavior according to

(4) $$\frac{d\,a^{\prime }}{d\,t^{\prime }}=\alpha \,\frac{A\,k^{\prime }}{Q+k^{\prime }}-\alpha \,a^{\prime }$$

In other words, *a*′ depends linearly on *k*′ for small *k*′, but approaches the saturation level *A* as *k*′ becomes large. The parameter *Q* represents the value of stiffness at which activity is half its maximum value.

Next, we want Eq. 2 to account for the homeostatic mechanism of enzymatic digestion of excess collagen in the case that tissue stiffness becomes too large, and conversely to allow for collagen deposition when stiffness becomes abnormally low. We thus define *g* such that *k*′ decreases when activity increases above a threshold *q*′, and increases when activity decreases below *q*′. This can be achieved by including a partitioning term (*a*′−*q*′) in *g* so that *g*(*a*′,*k*′) = (*a*′−*q*′)*h*(*k*′). To retain analytic solvability, we make *h*(*k*′) as simple as possible by giving it a linear dependence on *k*′ such that *h*(*k*′) = −*D*′*k*′, where *D*′ is a constant. These considerations lead to the rate equation for *k*′ being

(5) $$\frac{d\,k^{\prime }}{d\,t^{\prime }}=\left(a^{\prime }-q^{\prime }\right)\,\left(-D^{\prime }\,k^{\prime }\right)+P^{\prime }$$

where the constant *P*′ is a production term that serves to prevent (0,0) from being a fixed point of the system.

We now introduce the dimensionless variables for time *t* = α*t*′, activity $a=\frac{a^{\prime }}{A}$, and stiffness $k=\frac{k^{\prime }}{Q}$. Eqs 4 and 5 then become

$$\frac{d\,a}{d\,t}=\frac{k}{1+k}-a$$

$$\frac{d\,k}{d\,t}=\frac{A}{\alpha }\,\left(a-\frac{q^{\prime }}{A}\right)\,\left(-D^{\prime }\,k\right)+\frac{P^{\prime }}{\alpha \,Q}$$

Normalization also leads to a dimensionless digestion rate $D=\frac{A\,D^{\prime }}{\alpha }$, production rate $P=\frac{P^{\prime }}{\alpha \,Q}$ and activity threshold $q=\frac{q^{\prime }}{A}$, yielding the final system of model equations

(6) $$\frac{d\,a}{d\,t}=\frac{k}{1+k}-a$$

(7) $$\frac{d\,k}{d\,t}=\left(a-q\right)\,\left(-D\,k\right)+P$$

From Eqs 6 and 7 it is easily seen that if *P=0*, the fixed points are *a*^∗^ = *q*,  $k^{*}=\frac{q}{1-q}$. This has the undesirable attribute that the fixed point does not depend on the digestion rate *D*, and can be avoided by requiring that *P*≠0. Obviously, the threshold *q* at which cellular activity switches from production to digestion of ECM and the rate-constant *P* for production of ECM must also both be positive. Eqs 6 and 7, together with these constraints, thus constitute our model of self-healing that accounts for interactions between the cells that maintain the ECM and the stiffness of the ECM itself.

### Fixed Point and Its Stability

The fixed point of the system corresponds to the condition under which the left-hand sides of Eqs 6 and 7 are both zero. From Eq. 6, this gives $a=\frac{k}{1+k}$, which when substituted into Eq. 7 gives a quadratic equation for *k* having the two solutions

$$k_{1,2}=\frac{q\,D+P\pm \sqrt{{\left(q\,D+P\right)}^{2}+4\,D\,\left(1-q\right)\,P}}{2\,D\,\left(1-q\right)}$$

Since all model parameters are positive, and *q* < 1 (since *a* is always between 0 and 1), we have that $q\,D+P<\sqrt{{\left(q\,D+P\right)}^{2}+4\,D\,\left(1-q\right)\,P}$. This means that the only physically realistic (i.e., positive) stiffness solution for the fixed point is

(8) $$k^{*}=\frac{q\,D+P+\sqrt{{\left(q\,D+P\right)}^{2}+4\,D\,\left(1-q\right)\,P}}{2\,D\,\left(1-q\right)}$$

The corresponding activity fixed point is

(9) $$a^{*}=\frac{k^{*}}{1+k^{*}}$$

The stability of the fixed point is determined by the Jacobian $=\left(\begin{array}{cc} \frac{d\,f}{d\,a}\;\frac{d\,f}{d\,k} & \\ \frac{d\,g}{d\,k}\,\frac{d\,g}{d\,a} & \end{array}\right)=\left(\begin{array}{cc} -1\;\frac{1}{{\left(1+k\right)}^{2}} & \\ -D\,k-D\,\left(a-q\right) & \end{array}\right)$. The trace of this Jacobian is τ = −1−*D*(*a*−*q*) and the determinant is $\tau =D\,\left(a-q\right)+\frac{D\,k}{{\left(1+k\right)}^{2}}$. For stability, we need Δ > 0 and τ < 0 at the fixed point. The condition on Δ is that $D\,\left(a-q\right)+\frac{D\,k}{{\left(1+k\right)}^{2}}>0$, which gives $a^{*}>q-\frac{k^{*}}{{\left(1+k^{*}\right)}^{2}}$. The condition on τ is that 1 + *D*(*a*−*q*) > 0, which gives

(10) $$a^{*}>q-\frac{1}{D}$$

Substituting for *a*^∗^ from Eq. 9 into the condition on τ gives

(11) $$k^{*}>\frac{q\,D-1}{D-\left(q\,D-1\right)}$$

By setting *D* < 1, the right-hand sides of both Eqs 10 and 11 become negative, which guarantees stability. We used Eqs 8 and 9 to study the sensitivity of the fixed point to variations in the values of the model parameters. For time domain analysis, Eqs 6 and 7 were integrated using the ode23 solver of Matlab (R2018a, MathWorks, Natick, MA, United States).

### Computational Network Model

We implemented the analytic model on a computational network model of soft tissue. The network consisted of a hexagonal lattice of identical pre-stressed Hookean springs with fixed boundaries. At baseline (i.e., healthy), each spring had spring-constant *k*^∗^. Injury was simulated by setting the spring-constant of a single central spring to 2*k*^∗^. Self-healing of the network was simulated by placing a number of agents at random nodes (spring junctions) on the network and then allowing them to migrate from node to node with each time step, as in our previous model of pulmonary fibrosis (Wellman et al., 2018). Agent migration was either random or biased by local network stiffness. In the latter case, the probability of an agent moving to a given adjacent node was equal to the stiffness of the intervening spring as a fraction of the sum of the stiffnesses of all 3 springs impinging on the current node. The initial activities of all agents were set equal to *a*^∗^. At each subsequent time step, the activity level of every agent and the stiffness of any spring that an agent had just passed across were simultaneously updated according to Eqs 6 and 7, respectively.

The elastic equilibrium configuration of the network was computed after a pre-selected number of time steps by finding the node positions that minimized the total strain energy of the system using simulated annealing (Cavalcante et al., 2005). The stiffness of the network was obtained as the ratio of the change in average network stress when the network boundaries were varied biaxially by ±0.2%.

## Results

**FIGURE 1:**
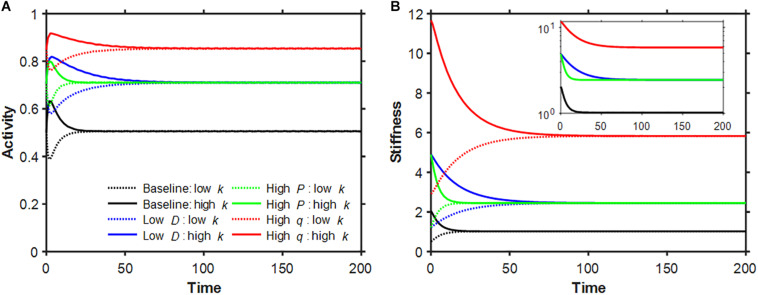
Time evolution of non-dimensionalized activity **(A)** and stiffness **(B)**. Baseline model parameters in these simulations were *q* = 0.2, *D* = 0.32 and *P* = 0.1 (black) corresponding to the dimensionless variables activity threshold, digestion rate and production rate, respectively. Additional simulations with low *D* (blue), high *P* (green) and high *q* (red) are also shown. The initial conditions were set to the fixed point in activity and half (solid dotted line) or double (solid line) the value of the corresponding stiffness fixed point. The inset plots the stiffness decays on a logarithmic scale. The dimensional parameters α,*A* and *Q* were set to unity.

**FIGURE 2:**
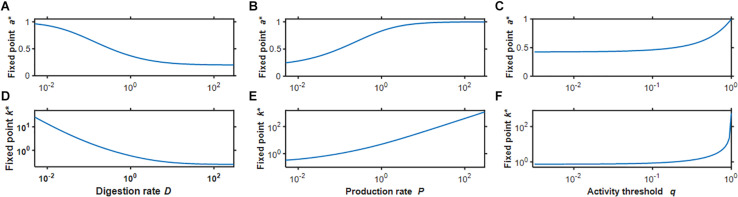
Fix points of activity **(A–C)** and stiffness **(D–F)** as a function of the 3 model parameters *D* **(A,D)**, *P* **(B,E)**, and *q* **(C,F)** corresponding to the dimensionless variables digestion rate, production rate, and activity threshold, respectively. Notice that stiffness is plotted on a double logarithmic scale.

Based on preliminary simulations, we chose the baseline parameter values *q* = 0.2,*D* = 0.32 and *P* = 0.1, which resulted in *a*^∗^ = 0.50559 and *k*^∗^ = 1.022612. Examples of the time evolution of activity and stiffness in the baseline model as well as following a 4-fold change in each model parameter are shown in Figure 1 when the initial value of stiffness was set to $\frac{k^{*}}{2}$ to represent tissue damage and 2*k*^∗^ to represent tissue scarring. In all cases activity asymptotes toward the fixed point, sometimes with a brief early excursion in the opposite direction. A 4-fold decrease in *D* shifted the fixed point by the same amount as a 4-fold increase in *P*; however, the approach toward the fixed point was slower in the low *D* case. Beyond the 100th time point each simulation was within 0.1% of the fixed point, consistent with a self-healing system. Interestingly, none of these time courses is exponential (Figure 1B inset) or a power law (not shown). Figure 2 shows how the fixed point varies with alterations in the model parameters. As *D* increases, both *a*^∗^ and *k*^∗^ decrease (panels A and D) whereas as *P* increases, *a*^∗^ exhibited a sigmoidal behavior and *k*^∗^ increased linearly on the log-log plot. When *q* was gradually increased, *a*^∗^ was constant first then approached unity while *k*^∗^ increased without a limit as *q*→1 in agreement with Eq. 8.

**FIGURE 3:**
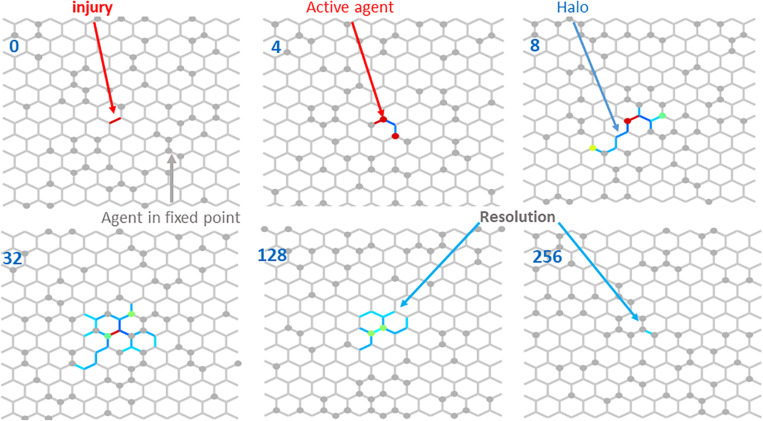
Resolution of an injury modeled as a single spring with a high stiffness (red line element) at iteration time 0 indicated in the top left corner. As unactivated agents (gray circles) visit the injury site, they become active (red circles at time 4) and after stepping off the injury site, they also stiffen the neighborhood creating a halo (defined as the set of spring with stiffess 0.1% higher than the fixed point) around the original injury site (time 8). Notice a yellow, a green, a red and several gray agents on or around the halo. Over the course of more time steps, the halo first increases (time 32), then gradually shrinks (time 128) and at time 256 there is only one network member left with a stiffness slightly higher than the fixed point. Eventually all members reach the fixed point. Colors from blue to red denote increasing agent activity and member stiffness. Gray members and agents represent fixed point values.

**FIGURE 4:**
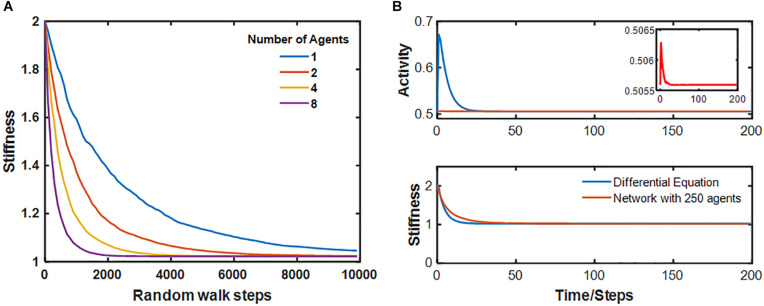
**(A)** Time course of stiffness decay of the central spring for several values of the number of agents in the network. **(B)** Comparison of the time course of the activity and stiffness of the network with 250 agents to that of the differential equation. For the network, the average agent activity is plotted. The inset shows a magnified region of the activity of the network.

Figure 3 illustrates the resolution of fibrotic injury in the network model. A single central spring was initialized to have *k* = 2*k*^∗^. A total of 64 agents were then allowed to migrate over the network, which consisted of 252 nodes and 345 springs, while Eqs 6 and 7 determined how the network member stiffness and agent activity evolved in time. At each time step the agents moved randomly to an adjacent node with a bias toward stepping over stiffer springs. By the 4th time step, two agents had visited the injury site where their activation levels became elevated. They then moved to neighboring sites and so raised the stiffness of the springs that they stepped over in the process. As this process continued, a “halo” of stiffer springs formed around the original injury site (time step 8). The halo grew initially (time step 32), but eventually resolved completely. The network model thus displays the property of self-healing. Figure 4A shows that the rate of self-healing increases with the number of agents involved in the healing process. Figure 4B compares the average activity of 250 agents and the corresponding stiffness decline of the single spring that had initial injury to the evolution of *a* and *k* predicted by the differential equation of the baseline model in Figure 1. Although the stiffness decline in the network is nearly identical to that of the differential equation, the average activity is very different. This is due to the large number of agents whose activity is still equal to the fixed point producing an average activity close to that of the fixed point. The inset, however, shows that the time course of the average activity is similar to that of the differential equation.

**FIGURE 5:**
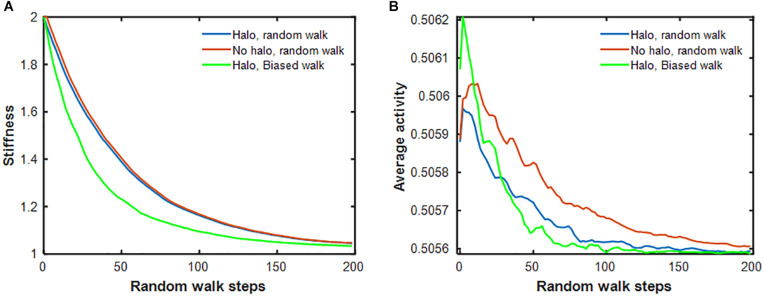
**(A)** Stiffness decay of the central spring for 3 different models: halo formation allowed during pure random walk (blue), halo formation prohibited during pure random walk (red) and halo formation together with stiffness-biased random walk (green). **(B)** Average activity of 64 agents during the same simulations as in panel **(A)**. The activities were smoothed with a running average filter.

**FIGURE 6:**
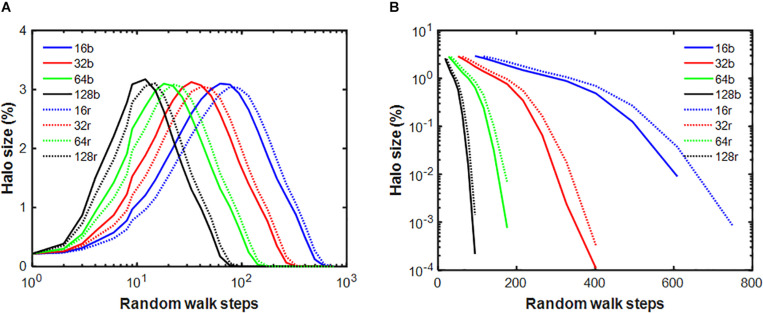
Evolution of the halo cluster size relative to the size of the network given in percent for the stiffness-biased (b: solid lines) and pure random (r: dotted lines) walks. Different colors represent different number of agents within the network. There is a statistically significant difference between the biased and random curves everywhere except the for smallest random walk step number. Panel **(A)** shows the random walk steps on log scale and panel **(B)** shows the halo size on log scale after the peak.

Next, we examined how the stiffness-biased agent migration and the presence of a temporary halo around an injury site influence the rate of self-healing. Figure 5A shows that the stiffness decay toward the fixed point produced by the pure random walk model (blue line) is significantly slower than that of the stiffness-biased walk (green line). We then eliminated halo formation by removing the ability of agents to modify spring stiffnesses, and instead imposing on the model the same stiffness decline in the central spring obtained from the pure random walk, while still updating agent activity at each time step. Figure 5B shows that activity was highest early on but then declined most rapidly with both stiffness-biased migration and the halo (green line), whereas the slowest decline is obtained with the pure random walk without a halo. To investigate the dynamics of halo formation, we repeated the stiffness-biased and pure random walk simulations 2,000 times and tracked halo size (defined as the number of springs for which *k* > 1.001*k*^∗^) in each simulation (Figure 6). Halo size increased initially to a peak value after which it slowly decreased for both the biased and random walks. However, the stiffness-biased walk reached the peak sooner and the halo also disappeared faster (the difference between the random and biased walks is highly statistically significant, *p* < 10^–7^). As more agents were working on the network, the curves shifted to the left completing the self-healing sooner. Figure 6B also demonstrates that the recovery phase is not a single exponential, and that increasing the number of agents yields faster halo resolution with less difference between random and biased walks.

**FIGURE 7:**
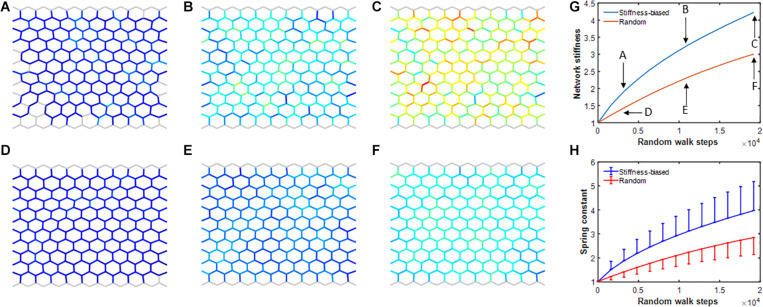
**(A–F)** Network configurations during simulations using the self-healing model on the network while agent activity was set to 0 after every second iteration. Top **(A–C)** and bottom **(D–F)** rows show networks in which agent walk was stiffness biased or pure random, respectively. Gray network members have normal stiffness of ∼1. Colors from blue to red indicate increasingly stiffer springs. **(G)** Network stiffness as a function of iteration number which represents time for stiffness-biased (blue) and pure random (red) walks. **(H)** Mean and SD of spring stiffnesses for stiffness-biased (blue) and pure random (red) walks.

To investigate how maintaining the system under sustained non-equilibrium conditions affects self-healing, agent activity was set to 0 (representing a highly active agent) after every second time step in the baseline model. Agents performed both a stiffness-biased or a purely random walk and the overall network stiffness as well as the mean and standard deviation (SD) of the individual spring stiffnesses were recorded. Figure 7 demonstrates that the biased random walk increased network as well as average member stiffness at a faster rate than the pure random walk. Comparing panels C and F in Figure 7, it is evident that the heterogeneity of the network is also noticeably higher in the stiffness-biased random walk model. Thus, the network model under sustained non-equilibrium conditions develops heterogeneously increased stiffness consistent with progressive fibrosis.

**FIGURE 8:**
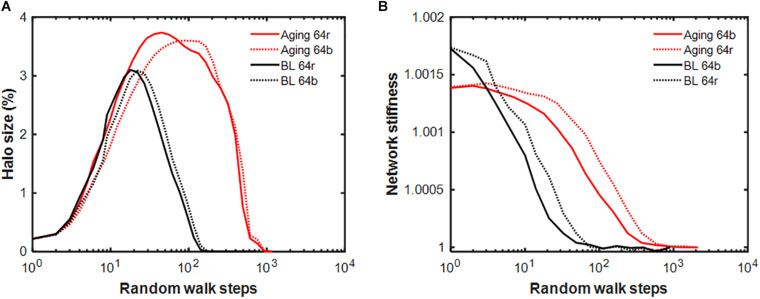
Comparison of the dynamics of network properties in the baseline and the aging models following an initial injury of setting a single spring constant to a value twice the fixed point. **(A)** Evolution of the halo size relative to the size of the network given in percent for the stiffness-biased (b: solid lines) and pure random (r: dotted lines) walks for the baseline (BL, black) and aging (red) models. **(B)** Relaxation of network stiffness normalized to the value after the injury has been removed. Notice that the magnitude of the network stiffness barely increases beyond the equilibrium value because less than 4% of the network members are affected by the halo.

Finally, the behavior of our network model has implications for healing in the context of aging, which is associated with increased tissue stiffness, weakened interaction between cells and tissues, reduced cell migration speed, and most importantly a reduction in the rate of self-healing in response to injury. To account for these features, we first increase the stiffness fixed point to *k*^∗^ = 2. From Eq. 9 we obtain $a^{*}=\frac{2}{3}$ which shifts the transition from ECM production to digestion to a higher level of agent activity. Setting the left-hand side of Eq. 7 to zero and using $a^{*}=\frac{2}{3}$ leads to $\frac{P}{D}=8/15$. Finally, substituting these into Eq. 7 and setting the left-hand side to zero results in $q=\frac{2}{5}$. According to Figure 1, lowering *D* slows the approach to the fixed point. Thus, we set *D* = 0.08 and from $\frac{P}{D}=8/15$, *P* = 0.0427, which guarantees no change in the fixed point. Using these parameters as a model of aging, we find significant changes in the halo dynamics (Figure 8A): compared to baseline, the halo reaches a larger cluster size with a peak that is delayed as computed from 2,000 repetitions of each random walk. Furthermore, elimination of the initial injury takes almost 10 times longer compared to baseline as the stiffness of the network also relaxes much slower in the aging model (Figure 8B). The total network stiffness computed from 20 repetitions is also sensitive to the type of random walk, demonstrating that the stiffness-biased walk further stretches out the self-healing time.

## Discussion

Recent advances in network theory and computational modeling have provided much insight into the two-way communication between sub-cellular, inter-cellular and integrated organ level mechanisms in physiological systems both in health and disease (Ivanov et al., 2016). The aim of this study was to investigate whether a computational model of cells communicating with their surrounding ECM network is capable of exhibiting behavior consistent with biological self-healing. We are not the first to attempt this, but previous studies have tended to focus on specific underlying processes or mechanisms (Huiskes et al., 2000; Taylor and Lee, 2003; Verberg et al., 2007; Bosia et al., 2014; Quattrociocchi et al., 2014; Shang, 2015), whereas our goal is to abstract the general principles of self-healing in their simplest form. To this end, we formulated an analytic model of the essential feedback process required for self-healing, and implemented it computationally in an elastic network representing the ECM of biological tissues. Our main findings are that: (1) properties of self-healing can be exhibited by dynamic non-linear interactions between only two variables, (2) when these interactions are incorporated into a spatially distributed network model of the ECM that is imbued with mobile agents, a halo of activity forms around a site of injury that attracts the agents and accelerates self-healing, (3) under sustained non-equilibrium conditions, the network model loses its capacity for complete self-healing and displays features of chronic progressive fibrosis, and (4) altering the strength of the interactions between agents and the network mimics the reduced rate of self-healing associated with aging.

We have identified three key attributes of a self-healing biological system, namely (1) automatic instigation of local reparative processes in response to injury, (2) sufficient material and energy reserves to complete the repair, and (3) cessation of the self-healing process when injury is resolved. The initiation of repair is inherent in the existence of the stable fixed point exhibited by Eqs 6 and 7, which causes the system to embark on a trajectory back toward “normality” when forced out of equilibrium (Figure 1). The availability of resources is not immediately evident from Eqs 6 and 7. In biology, a homeostatic fixed point (e.g., the stiffness of the skin or lung tissue) is an unlikely condition and according to the second law of thermodynamics, the order associated with the fixed point cannot be maintained unless external energy is utilized in the form of active maintenance. This, however, is not directly implemented in the model equations. Nevertheless, the equations represent a dissipative system due to the asymptotic approach toward the fixed point (Figure 1). Hence, the energy input associated with a given initial condition is dissipated during the return to the fixed point. Finally, the stability of the fixed point guarantees that reparative processes asymptotically approach zero as the system approaches its baseline equilibrium state. Of course, homeostasis in a real biological system is characterized by fluctuations within a local region of state space (Suki et al., 2020), so the single fixed point in our model corresponds to being within this region. Therefore, within the limitations imposed by its extreme simplicity, the dynamical system described by Eqs 6 and 7 displays the key features of self-healing.

Self-healing over the spatial extent of the network implementation of the model (Figure 3), requires that agents are able to reach every node of the network. If the agents are stationary, then a large number of agents is required so that an agent can be positioned ready to act at every node. Economically, this would require a large chunk of the total energy available for an organism. Far fewer agents are required if they have the ability to move around in the network, particularly if their movements can be directed to sites where agent activity is required. Nature’s solution to this question is to have key reparative cells, such as fibroblasts, continually migrating around within a tissue, and to be responsive to gradients in appropriate chemical or biological signals so as to home toward sites of injury. Accordingly, we implemented repair in our network model via a relatively small number of mobile agents whose behavior (their activity and preferential directions of movement) is influenced by the nature of the network (its local stiffness). The rate of repair of a damaged network depends, not surprisingly, on the concentration of the agents (Figure 4A). The dynamics of self-healing become similar to those of Eqs 6 and 7 as the number of agents approaches the number of nodes in the network (Figure 4B), but at lower agent concentrations we see the emergence of a new spatiotemporal phenomenon resulting from agent-network interactions. These interactions also allow agents to indirectly influence each other’s behavior; if an activated agent increases the stiffness of a given spring by passing over it, when this spring is subsequently visited by a second agent the activation level of the second agent will also increase. In other words, agent-agent communication emerges from spatial interactions of individual agents with the underlying ECM network. Furthermore, this communication causes the changes in ECM stiffness to become spatially correlated, giving rise to the halo effect (Figure 4), as agents carry information about local stiffness to surrounding regions. Intriguingly, this emergent behavior results in more efficient self-healing (Figure 5).

The halo effect (Figure 3) is reminiscent of how a physiological state is related to the underlying network dynamics (Bashan et al., 2012). Specifically, the halo describes the spatiotemporal evolution of inflammation that first grows around an initial injury site and then decays away as healing approaches completion. The same dynamics are evident in the halo (Figure 6). These dynamics coincide with the time decay of the injury itself (Figure 5A), and are significantly affected by cell-ECM interactions. For example, if the agents adhere to a stiffness-biased random walk, the spread of the halo peaks sooner, and both the halo and the initial injury resolve more quickly compared when there are no cell-ECM interactions in the case of a pure random walk (Figure 6A). Interestingly, when the time variation of halo size is plotted on a time scale normalized by the inverse of agent numbers, the halo dynamics become nearly independent of agent numbers (Figure 6B). The dynamics therefore result from the rate of local self-healing characterized by how fast the system approaches the fixed point relative to the speed of agent migration.

The existence of the halo demonstrates that our model exhibits a process known as durotaxis in which directed migration of cells is influenced by substrate stiffness (Lo et al., 2000). Our model results further demonstrate that durotaxis contributes to the efficiency of self-healing (Figure 5), suggestive of its evolutionary advantages. However, simulating sustained exposure to a noxious stimulus in the face of impaired reparative function (by periodically setting agent activity to zero representing highly active agents) prevents the network model from returning to equilibration after injury. Furthermore, durotaxis accelerates aberrant tissue remodeling (fibrosis formation) by providing a positive feedback through which cells preferentially migrate to stiffer members, which in turn increases the overall stiffness of halos. Indeed, comparing the images in Figures 7C,F, it is evident that durotaxis increases stiffness in the neighborhood of hot spots (red-colored members), which leads to a higher overall network stiffness.

The structural alterations that take place in fibrotic tissue reflect both excess collagen deposition (Kirk et al., 1986) and enhanced cross-linking (Reiser et al., 1986). Of these, dysregulated collagen cross-linking resulting in structurally and functionally abnormal collagen fibrils may bear most of the responsibility for the elevated stiffness seen in fibrosis (Jones et al., 2018). Imbalances in anabolic and catabolic processes are also a fact, however. For example, matrix metalloproteinase-1 (MMP-1), the key enzyme responsible for collagen digestion, was found to be significantly decreased and its inhibitor increased in a rat model of pulmonary fibrosis (Hu et al., 2015). These pathological changes can be represented in our network model by reducing *D* and increasing *P*, both of which elevate the fixed point in stiffness (Figure 1). In contrast, having agents perform a stiffness-biased random walk, to mimic how fibroblasts are activated on stiffer ECM (Asano et al., 2017) with a directionality toward stiffer regions (Lo et al., 2000), alters the dynamics of self-healing but not the value of the fixed point. These dynamics are accelerated during repair of a single isolated injury (Figure 6), but also accelerate the formation of chronic fibrosis in the face of a chronic insult (Figures 7G,H). We recently used an agent-based model to show that cell-ECM interactions can give rise to the characteristic honeycomb features seen in the lung periphery in pulmonary fibrosis (Wellman et al., 2018). The model of the current study complements this finding by showing that cell-ECM interactions can also contribute to the development of fibrosis in the presence of misguided self-healing due to the sustained application of non-equilibrium forces by an environmental insult.

Finally, our model also suggests insights related to the changing self-healing of biological tissue as an organism ages. When the parameter *q* is increased to reflect an elevation in the threshold at which agent activity switches between production and digestion, the stiffness fixed point increases and the rate of self-healing decreases. The same effect occurs when either the ECM digestion rate (*D*) or production rate (*P*, not shown) are reduced from their baseline values (Figure 1). The corresponding halo dynamics are distinctly different from the baseline (Figure 8A) suggesting that changes in internal signaling affect the spatial extent of the inflammatory response to injury as well as its resolution (Figure 8B). Reductions in *D* and *P* are biologically consistent with aging. For example, both the turnover of collagen (Pierce et al., 1967; Mays et al., 1991) and the activity of lysyl oxidase, a key extracellular enzyme in collagen and elastin processing, decrease with age (Poole et al., 1985). Additionally, β1 integrin, which plays a key role in ECM stiffness sensing (Gershlak and Black, 2015), is downregulated in aged human skin (Giangreco et al., 2010). Since integrins physically link cells to the ECM in order to allow bi-directional signaling (Hynes, 2002), the loss of integrins can be interpreted as decoupling the cell from the ECM in aging. In our model, ECM sensing by cells is represented by the stiffness-biased random walk of the agents. When this sensing is reduced in the model, the halo size further increases (Figure 8A) and the rate of self-healing becomes slightly slower (Figure 8B).

The simple model presented here has many limitations. First, the differential equations (Eqs 6 and 7) neglect all intracellular structures and processes as well as different cell types. Indeed, stable two-cell systems including macrophages and fibroblasts displaying cell-cell contacts are essential to homeostasis (Zhou et al., 2018). Our model utilized a single effective agent and we focused on its interaction with the ECM. The various cytokines and cell types of the immune system are known to contribute to self-healing locally (Brazzola et al., 2003; Anand and Tiwary, 2010; de-Campos et al., 2010; Wang et al., 2013) and to wound healing (Wilson et al., 2001). These responses are implicitly lumped together in our model as the single agent activity level, but this precludes the possibility of representing the individual relative roles of the different players, many of which can significantly modulate the time course of the healing process (Ohshima and Sato, 1998; Kato et al., 2011). Also, the network model is only a 2-dimensional system without the spatial heterogeneity of structure that is characteristic of real tissues, nor does it contain ancillary structures such as airways that can contribute to pulmonary fibrosis (Miller et al., 2019). Specific bond ruptures and physical self-healing by reforming bonds in the network, as in cross-linked nanogels, have been neglected (Duki et al., 2011). Although a previous model of chondrocytes included cellular random walk, cell behavior was not connected to tissue healing (Vaca-Gonzalez et al., 2017). In the present study, the interaction between the ECM and the cells is also greatly simplified. For example, although we allowed for durotaxis, no intracellular mechanisms are associated with it, and cellular migration itself is either purely random or biased walk based on only the single factor of stiffness. The processes associated with aging are also extremely complex. For example, aging may slow or completely block cell migration following injury (Moraga et al., 2015), which may further slow the self-healing dynamics, yet we considered only the removal of bias from cell migration as a consequence of aging in our model. Finally, in aiming to develop the simplest possible conceptual model capable of capturing the essence of self-healing, we have limited ourselves to a dynamic system with only a single fixed point. An interesting, but somewhat more complicated possibility for aberrant healing arises in systems with more than one fixed point in which an interaction with the environment may potentially push the system into the basin of attraction of a different attractor that represents chronic disease (Anafi and Bates, 2010). In the context of a two-cell system including macrophages and myofibroblasts, bi-stability of the circuit can help explain how stimulus magnitude, duration and its repetitive nature leads to fibrosis (Adler et al., 2020). While our analytic model neglects these details, the network approach allows us to explore the spatial aspects of the healing or fibrosis dynamics. Future work should investigate how a more detailed network-agent model is able to account for the specific cellular and macroscopic features of the tissue during the evolution of self-healing.

In conclusion, we have developed a computational model that exhibits three key properties necessary for successful self-healing. The model includes the non-linear interactions between two essential variables, agent activity and stiffness, imposed on a distributed elastic network. The model recapitulates the growth and resolution of inflammation and repair around a site of tissue injury, and shows how misguided self-healing leading to fibrosis can arise in the presence of a persistent external irritant, and how factors related to aging can impair and retard the healing process.

## Data Availability Statement

The raw data supporting the conclusions of this article will be made available by the authors, without undue reservation.

## Author Contributions

BS developed model, carried out computations, and wrote manuscript. JH and JB developed model and wrote the manuscript. All authors contributed to the article and approved the submitted version.

## Conflict of Interest

The authors declare that the research was conducted in the absence of any commercial or financial relationships that could be construed as a potential conflict of interest.
